# 

**Published:** 1880-09

**Authors:** 


### CONTENTS FOR SEPT'B'R, 1880.

ORIGINAL COMMUNICATIONS.

ART. I.—Some Practical Suggestions in the Treatment of Diphtheria. By R. J. NUNN, M. D. .... 415
ART. II.—Some suggestions in regard to the treatment of Gonorrheal Rheumatism. By WM. A. BYRD, M. D. .... 422
ART. III.—Dental Education. By Dr. GEO. H. PERINE .... 425
ART. IV.—Case of Purpura Hemorrhagica resulting in Dropsy. By DUDLEY M. CULVER, M. D. .... 426
ART. V.—Our Duty. By DR. J. ALLEN OSMUN, 427
ART. VI.—Treatment of Fracture of the Jaw, with critical remarks. By. THOMAS BRIAN GUNNING, D. D. S. .... 430
ART. VII.—The Ethnological Phenomenon. By J. C. BECKHAM, M. D. .... 438
Correspondence .... 439

EDITORIAL DEPARTMENT.

City Hospital .... 440
Trommer's Extract of Malt .... 442
Lactopeptine .... 442
Medical Fees and Under Bidding .... 443
A Baltimore Letter .... 444
Mortuary and Thermometrical Reports .... 448
Seats for Female Attendants in stores .... 448
The Chief Diploma Vender gone .... 448
Bibliographical Department .... 449
Hymeneal, Necrological .... 455

EXCERPTA.

The Evolution of Consciousness .... 455
Female Physicians in the Olden Time .... 459
Death of George Washington .... 459
The Tanner Fast .... 460
Prevention of Lacerated Perineum .... 460
Treatment of Anal Fissure .... 461
Bromide of Potassium .... 461
Mortuary Statistics .... 462

